# Transactional sex and age-disparate sexual partnerships among adolescent girls and young women in Tanzania

**DOI:** 10.3389/frph.2024.1360339

**Published:** 2024-07-11

**Authors:** Katherine B. Rucinski, Gaspar Mbita, Kaitlyn Atkins, Esther Majani, Albert Komba, Caterina Casalini, Mary Drake, Neema Makyao, Anthony Galishi, Yeronimo Mlawa, Joyce Wamoyi, Sheree Schwartz, Stefan Baral, Kelly Curran

**Affiliations:** ^1^ Department of International Health, Johns Hopkins School of Public Health, Baltimore, MD, United States; ^2^ Jhpiego, Monrovia, Liberia; ^3^ Athena Institute, Vrije Universiteit, Amsterdam, Netherlands; ^4^ Department of Epidemiology, Johns Hopkins School of Public Health, Baltimore, MD, United States; ^5^ Embassy of Switzerland in Tanzania, Dar es Salaam, Tanzania; ^6^ Jhpiego, Dar es Salaam, Tanzania; ^7^ Amref Health Africa, Dar es Salaam, Tanzania; ^8^ National Malaria Control Program, Ministry of Health, Dodoma, Tanzania; ^9^ USAID Tanzania, Dar es Salaam, Tanzania; ^10^ National Institute of Medical Research, Mwanza, Tanzania; ^11^ Jhpiego, Baltimore, MD, United States

**Keywords:** AGYW, HIV prevention, epidemiology, transactional sex, older partners

## Abstract

**Introduction:**

Adolescent girls and young women (AGYW) continue to experience a high incidence of HIV in southern and eastern Africa, even in the context of large-scale HIV prevention interventions. In Tanzania, AGYW account for the largest proportion of new infections and have a higher risk of HIV acquisition than males of comparable age.

**Methods:**

We used routinely collected data from the PEPFAR/USAID-funded Sauti Project, a large combination HIV prevention program, to examine the relationship between transactional sex and sex with older partners among AGYW in Tanzania (2015–2020). Out-of-school AGYW 15–24 years completed a vulnerability index and were tested for HIV. We estimated weighted prevalence ratios (PR) and 95% confidence intervals (CI) for the associations of transactional sex (sex exchanged for money/services/gifts outside of sex work) and sex with older partners (≥5-years older, ≥10-years older) with prevalent HIV. Age cutoffs of 5 and 10 years were used to align partner age differences with age-disparate and intergenerational sex, respectively. We assessed potential synergism between exposures, and subgroup analyses explored associations among girls 15–19.

**Results:**

Sixty seven thousand three hundred fifty seven AGYW completed the vulnerability index and 14,873 had captured HIV testing records. Median age was 20 years (IQR 18–22). Transactional sex and age-disparate sex were common (35% and 28%, respectively); 13% of AGYW reported both behaviors. HIV prevalence was associated with both transactional sex (PR: 1.28; 95% CI 1.00–1.63) and age-disparate sex (PR:1.26, 95% CI 0.99–1.60). In common referent analysis, transactional sex remained strongly associated with HIV, even in the absence of age-disparate sex (PR 1.41; 95% CI 1.02–1.94).

**Discussion:**

Evidence of statistical synergism was not present, suggesting both transactional sex and age-disparate sex operate through similar pathways to increase HIV risk. Increased specificity within HIV prevention programs is needed to better meet the sexual and reproductive health needs of AGYW at high risk of HIV in Tanzania, including investment in tailored youth-friendly strategies for AGYW who have been marginalized from the current HIV response.

## Introduction

Over the last decade, substantial global investment and the expansion of critical prevention and treatment programs have resulted in overall declines in HIV incidence in countries across sub-Saharan Africa, including in Tanzania (1, 2). Despite this progress, the burden of HIV remains high among key and other vulnerable populations such as adolescent girls and young women (AGYW) ages 15–24 (3). In 2022, AGYW account for the highest proportion of new infections in Tanzania and remain at greater risk of infection relative to their male counterparts and other age groups (1, 4, 5).

Structural, economic and social dynamics in eastern and southern Africa, along with increased biological vulnerability, heighten young women's risk of acquiring HIV (6, 7). During adolescence and early adulthood, key transitions including physical, physiological, emotional, and social changes happen rapidly, affecting relationship formation and sexual decision making (8–11). As young people navigate these changes, gender norms that assign greater social and economic power to men create the material and ideological conditions that make it difficult for young women to refuse sex, negotiate condom use, or advocate for their own sexual interests (12–15). These dynamics may be heightened for young women living in poverty, including AGYW that live in food-insecure households and are unable to readily meet their basic needs from sources other than boyfriends or other sexual partners (16).

Sexual relationships with older male partners can increase the risk of HIV for AGYW in eastern and southern Africa. These relationships, which often comprise a sexual relationship with a male partner ten or more years older, can also lead to transmission of other sexually transmitted infections, early pregnancy, and child marriage (17, 18). In South Africa, increasing differences in partner age are associated with risk of HIV infection, particularly for adolescents (19). Phylogenetic studies also indicate that men 25–40 years are the main source of HIV transmission for AGYW ages 15–25 (20). Similar findings have emerged in Kenya, Uganda, and Zimbabwe, with partner age differences as little as five years predictive of elevated HIV risk (21, 22).

Transactional sex, defined by the exchange of sex for money or material support, has also been shown to be consistently associated with HIV acquisition (23–25). Transactional sex exists across a continuum for AGYW, with exchanges occurring within established partnerships, casual encounters, or within the context of more formalized sexual exchanges such as sex work (26, 27). AGYW who report transactional sex have also reported a high prevalence of emotional and sexual violence, substance use, and condomless sex. When transactional sexual relationships occur with older males, these HIV risks can be further amplified given a higher prevalence of infection (28–30).

Study of transactional sex with older partners among AGYW in eastern and southern Africa has increased over the last five years, with inferences largely made using established research cohorts in countries such as South Africa, Uganda, and Kenya (23, 31, 32). While substantial qualitative work has been done to contextualize experiences of transactional sex in Tanzania, few empiric studies have quantitatively examined the relationship between transactional sex and HIV in the context of age-disparate relationships (22, 27, 33, 34). Here, we use routinely collected data from a large combination HIV prevention program to better define the relationship between transactional sex and sex with older partners, and their joint impact on the HIV burden among AGYW in Tanzania.

## Methods

### Study setting and program

**Figure 1:**
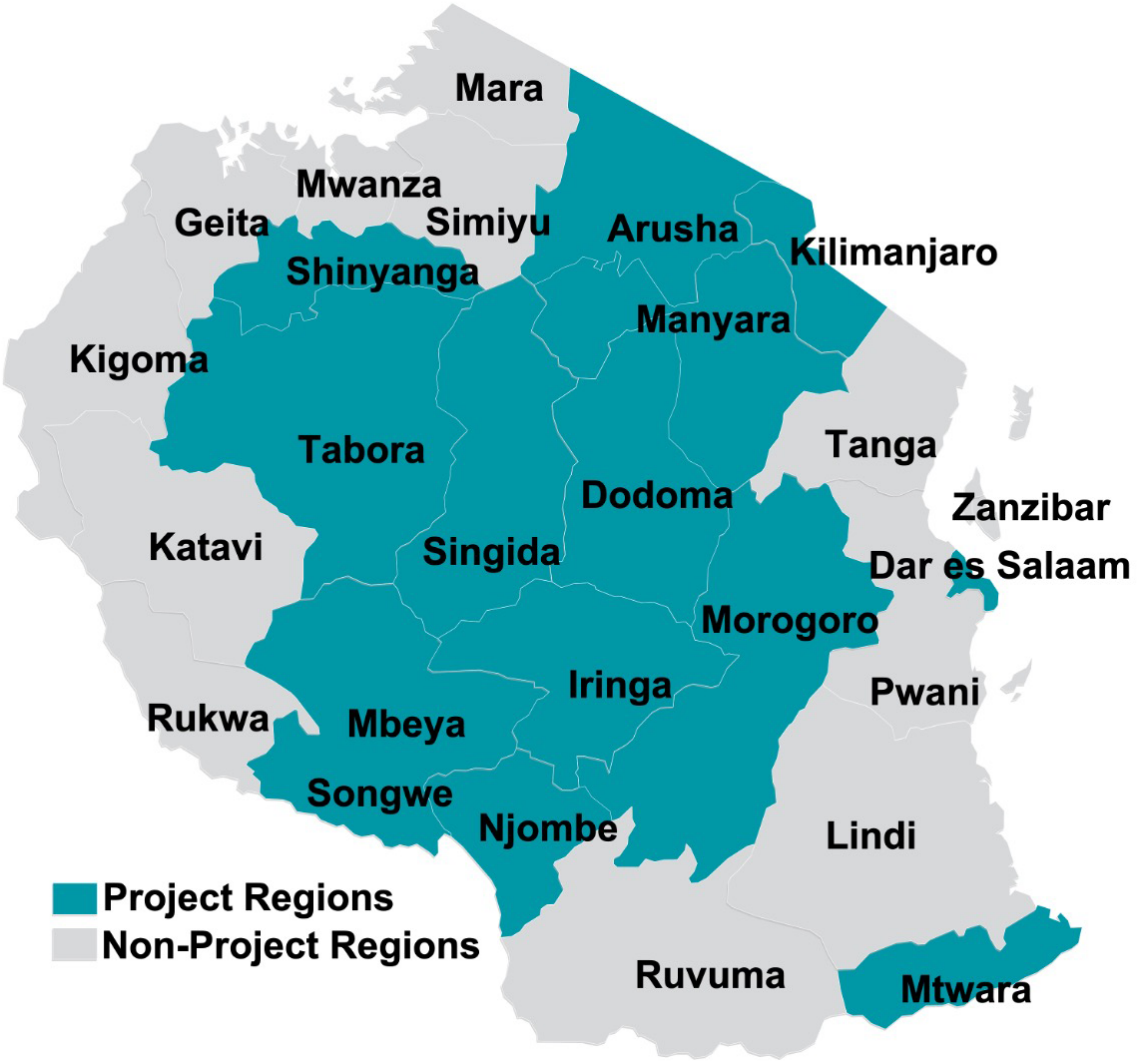
Sauti project regions in Tanzania, 2015–2020.

Data included in this analysis are from the Sauti Project, a large community-based HIV prevention program funded by the United States Agency for International Development (USAID) through the President's Emergency Plan for AIDS Relief (PEPFAR). Implemented by Jhpiego in partnership with EngenderHealth, Pact, and the Tanzania National Institute for Medical Research, Sauti provided a core package of client-centered sexual and reproductive health services for key populations such as female sex workers and men who have sex with men (35–39). Sauti also delivered critical HIV-prevention services for vulnerable AGYW ages 15–24 within 14 regions of Tanzania between 2015 and 2020, and was also an implementing partner of the DREAMS (Determined, Resilient, Empowered, AIDS-free, Mentored, and Safe) Initiative (Figure 1) (34, 40). Programming delivered through Sauti for AGYW included biomedical services such as HIV testing and counseling, HIV case management, sexual risk assessments, family planning counseling and provision of contraception, screening for sexually transmitted infections (STI) and tuberculosis, nutritional assessments, and additional screening and referrals for substance use and gender-based violence. Further programming included behavioral change sessions and social protection interventions such as savings and loans clubs.

### Recruitment, eligibility, and procedures

AGYW between the ages of 15 and 19 who were not in school full time (defined as not having attended classes more than 10 days in the last three months when not on public holidays or school vacation) and those aged 20–24 were recruited beginning in 2015. Venues included biomedical service-delivery points such as community-based HIV testing and counseling sites, workplaces, bars, guest houses, salons, markets, other venues previously identified as “hotspots” by the program, or AGYW-identified DREAMS safe spaces including religious houses and local government offices. Trained peer facilitators recruited AGYW from each site and assessed their eligibility for program services using a brief tool. This vulnerability index was adapted using existing tools from Tanzania and other countries in eastern and southern Africa (40). Methods to develop this index have been previously described (40). Briefly, measures included basic demographics, indicators of sexual experience including partner number and age, engagement in transactional sex, experiences of intimate partner violence, depression, and food insecurity, among others. The full index is reported in the (Supplementary File S1). Further, AGYW who accessed HIV testing and other biomedical services through Sauti also completed a health screening and service tool (HSST). Information routinely collected through the HSST included basic demographics, screening for harmful drinking of alcohol and substance use, STI screening and treatment, and each AGYW's HIV testing results. Typically, these HIV testing visits occurred after AGYW had engaged in behavior change sessions, economic empowerment sessions or other Sauti biomedical services.

### Study population

We assembled an analytic sample using data collected among AGYW through the vulnerability index and merged these data with biomedical HIV testing records collected through the HSST using participant-specific alphanumeric codes. Data were restricted to those AGYW that accessed services through Sauti between 2015 and 2019, and to those AGYW that were eligible for inclusion based on the established eligibility criteria as described. Notably, some AGYW who did not meet full eligibility requirements, including those that were still in school, were also referred for Sauti services if they expressed interest in engaging with the program; these AGYW were excluded from analyses. In some cases, AGYW had duplicate or repeat vulnerability index records. For these AGYW, we used the earliest record available in the database. Similarly, some AGYW had multiple HIV testing results reported over multiple years. For these AGYW, we used the date of first HIV testing record under the assumption that this visit occurred closet to initial program engagement. Young women ages 18–24 who self-identified as female sex workers were also excluded from analyses, as they were ineligible to complete the AGYW vulnerability index through Sauti.

### Outcome and exposures

The primary outcome in this analysis was prevalent HIV infection, defined as a positive HIV test result documented through the Sauti HSST database. Standard HIV testing comprised serial testing using the SD Bioline HIV-1/2 3.0 (Standard Diagnostics Inc., Suwon, Korea) and Uni-Gold HIV-1/2 (Trinity Biotech, Wicklow, Ireland) assays. Mode of transmission was not assessed, and we were unable to discern between sexually and pediatrically acquired infections.

The two primary exposures included in this analysis were self-reported engagement in transactional sex and sex with older partners, both assessed through the vulnerability index. Transactional sex was defined as having ever engaged in any sex for money, services, or gifts (yes/no) (26). Age-disparate sex was defined as having engaged in sex with a partner five or more years older; intergenerational sex was defined as having ever engaged in sex with a partner ten or more years older (yes/no) (41, 42).

### Statistical analysis

Demographics and characteristics of AGYW participants who completed the vulnerability index were summarized using proportions for categorical variables and medians for continuous variables. Chi-square tests were used to compare the differences in proportions between groups for categorical variables, and Wilcoxon signed-rank sum tests were used for continuous data (alpha = 0.05).

We assessed differences in sample characteristics among participants who did and did not have a linked HIV testing result confirmed through the HSST. Among participants with an HIV testing result, we further assessed differences in history of transactional sex, comparing those who had engaged in transactional sex with those who reported never having engaged in transactional sex. We used a modified Poisson regression model with a robust variance estimator to estimate prevalence ratios (PR) and 95% confidence intervals (CI) for the association between transactional sex and prevalent HIV infection. Models were restricted to AGYW who were sexually active. A directed acyclic graph (DAG) was used to identify a minimally sufficient set of confounders for adjustment. Covariates in the minimally sufficient set included age, age-disparate sex/intergenerational sex, adult support, marital status, prior pregnancy, food insecurity, year, and early sexual debut. Models were similarly developed to assess associations between age-disparate sex and HIV, as well as intergenerational sex and HIV. Interaction was assessed between transactional sex and age-disparate sex, and between transactional sex and intergenerational sex using common referent analysis and through comparison of observed and expected joint effects (43). Subgroup analyses assessed main associations among adolescent girls ages 15–19.

### Weights

Given missing HIV testing outcomes among those AGYW that did not have a linked record through the HSST, we derived non-response weights under the assumption that these data were missing at random (MAR) to address the possibility of selection bias and to improve the internal validity of our findings (44, 45). Weights were created using the full database of 67,357 unique and eligible AGYW participants who completed the vulnerability index. An indicator variable for having an observed vs. a missing HIV testing result was created and estimated as a function of a participant's measured covariates including age, partner status, prior pregnancy, food insecurity, sexual debut, experiences of violence, region, and survey version (year). Covariates were chosen based on preliminary analyses to identify patterns of missing information in these data, and we used multiple imputation by chained equations (MICE) to fill in missing data for all covariates (*n* = 10 imputations) (46, 47). We calculated and applied weights to all effect estimates for each series of imputations. Weights were stabilized by the marginal probability of having an observed HIV testing outcome and were truncated at the 5th and 95th percentiles to further improve stability. Weighted effect estimates and standard errors were pooled to calculate final PRs and 95% CIs, standardized to the full population of AGYW who completed the vulnerability index.

All analyses were conducted using SAS 9.4 (SAS Institute, Cary, NC).

### Ethics

The National Institute for Medical Research and the Ministry of Health Community Development, Gender, Elderly and Children of the United Republic of Tanzania provided ethical clearance for primary data collection activities under Sauti, including both primary and health program screening data in the context of routine service delivery. The vulnerability index was administered as a programmatic tool to support quality improvement in the delivery of differentiated HIV prevention services for AGYW in Tanzania. All participants provided verbal informed consent given extremely low literacy rates in the population. Parental consent was only sought for HIV testing if the participant was under the age of 18 in accordance with Tanzania law. Minors under the age of 18 who were parents were considered emancipated, and thus did not require parental consent. Ethical approval for the use of de-identified routine data was provided by the National Institute for Medical Research (NIMR/HQ/R.8c/Vol.1/678) and the Johns Hopkins Institutional Review Board (IRB No. 00006673).

## Results

### Sample characteristics

**Figure 2:**
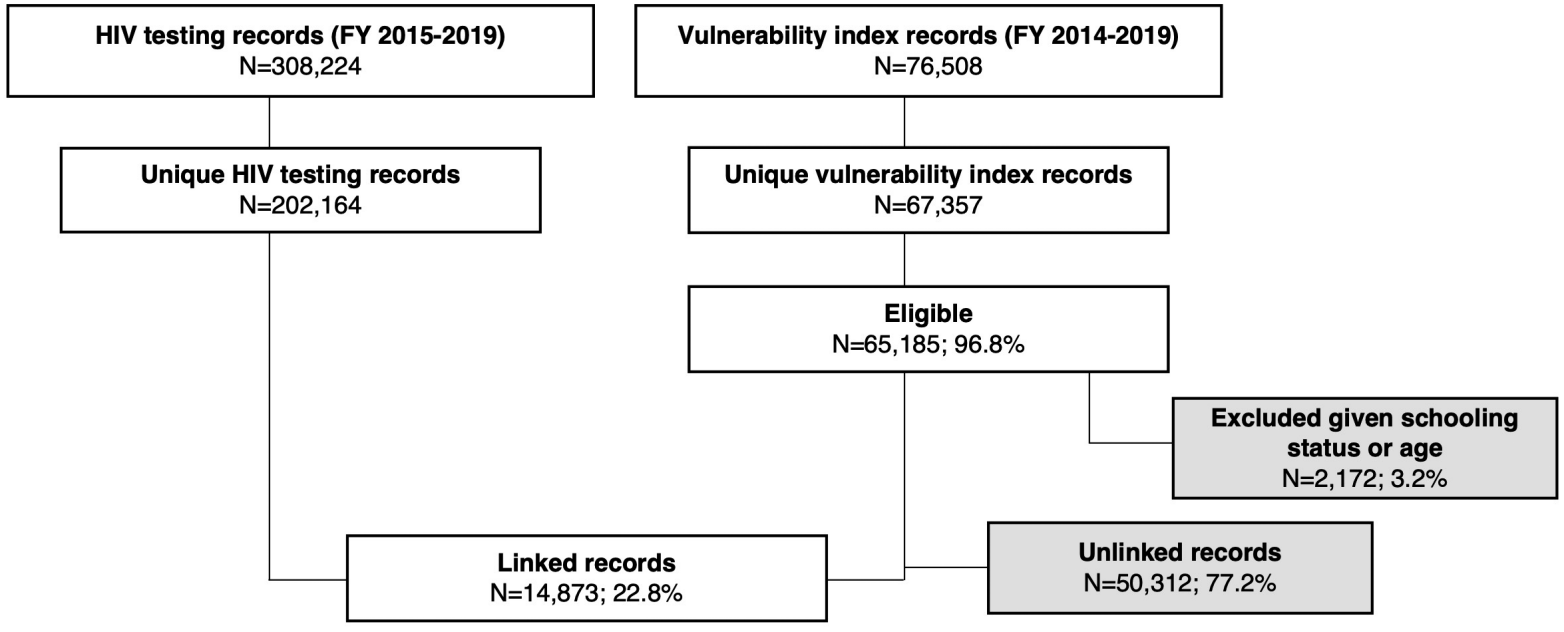
Construction of analytic sample including 14,873 AGYW using program data from the sauti project, 2015-2019.

A total of 67,357 unique AGYW participants completed the Sauti vulnerability index between 2015 and 2019 (Figure 2). Based on their age and schooling status, 65,185 were eligible for inclusion and retained in further analyses. Of these AGYW participants, 19,748 (30.4%) lived in Dar es Salaam, 11,240 (17.3%) in Mbeya, and 8,891 (13.7%) in Shinyanga; the remaining participants were from Arusha, Dodoma, Iringa, Kilimanjaro, Morogoro, Njombe, Songwe, and Tabora.

A total of 14,873 (22.8%) AGYW were able to be linked to their HIV testing record through the HSST using a unique identifier. Of these 14,873 AGYW, the median age was 20 years (IQR 15, 24) (Table 1). One-fifth (20.9%) were married. Almost half (40.7%) had previously been pregnant. Lifetime experience of transactional sex and age-disparate sex were common (35.6% and 39.1%, respectively); 13% of AGYW reported both behaviors. A total of 278 (1.9%) AGYW were living with HIV. Characteristics differed significantly by experience of transactional sex.

**Table 1:** Characteristics of 14,873 AGYW accessing combination HIV prevention services in Tanzania through the Sauti project, 2015–2019^a^.

	Overall *N* = 14,873		No transactional sex *N* = 9,521 (65.4%)		Transactional sex *N* = 5,047 (34.6%)		
	Median	IQR	Median	IQR	Median	IQR	*p*-value
Age, years	20.0	18.0–22.0	20.0	18.0–22.0	21.0	19.0–23.0	<.001
** **	*n*	%	*n*	%	*n*	%	*p*-value
Married	3,063	20.9	1,916	20.5	1,096	22.0	<.001
Adult support^b^	7,949	55.0	4,381	47.1	3,482	70.2	<.001
Food insecurity^c^	4,302	29.0	2,081	22.3	2,179	44.4	<.001
Ever pregnant	6,035	40.7	3,369	35.4	2,587	51.3	<.001
Sexual debut <15 years	2,544	17.7	1,099	11.8	1,412	28.7	<.001
Sexual partner >5 years older	4,162	28.3	2,156	22.8	1,953	39.1	<.001
Sexually active, ever	12,730	86.4	7,509	79.0	5,006	99.9	<.001
Sexually active, last 12 months	10,895	76.1	6,230	67.0	4,522	92.9	<.001
Using modern contraception^d^	4,774	36.8	2,663	31.5	2,111	46.5	<.001
Condomless sex, last 12 months^e^	791	74.1	472	68.0	319	85.5	<.001
>1 partner, last 12 months	5,014	34.8	1,777	19.0	3,127	65.4	<.001
Sexual violence, ever	1,901	13.1	771	8.2	1,109	22.4	<.001
Sex with HIV + partner, last 12 months	411	2.9	186	2.0	216	4.4	<.001
Prevalent HIV	278	1.9	150	1.6	125	2.5	.006

^a^ Missing. transactional sex: 305 (2.1%); married: 244 (1.6%); adult support: 427 (2.9%); food insecurity: 484 (3.3%); pregnancy: 25 (0.2%); sexual debut: 470 (3.2%); partner age: 153 (1.0%); ever sexually active: 547 (3.7%); sexually active in the last year: 139 (0.9%); contraception: 659 (4.8%); number of sex partners: 438 (2.9%); sexual violence: 390 (2.6%); sex with HIV + partner: 438 (2.9%); HIV: 341 (2.3%).

^b^ Has an adult in the household or community who provides unconditional emotional and/ or financial support.

^c^ Could not afford to buy food or there was not enough food to eat at home.

^d^ Use of modern contraception asked on versions 1 and 2 of the index.

^e^ Used a condom never, almost never, or sometimes when having vaginal sex; asked on version 3 of the index.

The remaining 50,312 participants did not have a matching HIV testing record in the database. Compared to those for whom an HIV testing record was not available, AGYW with observed HIV testing outcomes were less likely to be married (20.9% vs. 23.6%; *p* < 0.001), to have experienced recent food insecurity (29.9% vs. 40.8%; *p* < 0.001), to have ever experienced sexual violence (13.1% vs. 21.8%; *p* < 0.001), and to have engaged in transactional sex (37.0% vs. 45.1% *p* < 0.001). Characteristics among AGYW participants with and without a linked HIV testing record are reported in Supplementary Table S1.

### Transactional sex and HIV

Lifetime experience of transactional sex was associated with HIV prevalence in unadjusted (PR: 1.41; 95% CI 1.10, 1.80) and adjusted models (PR: 1.34; 95% CI 1.04, 1.74) (Table 2). Weighted estimates similarly demonstrated a population-level association between transactional sex and HIV (PR: 1.28; 95% CI 1.00, 1.63).

**Table 2:** Unadjusted, adjusted, and weighted associations of (A) transactional sex with HIV prevalence; (B) age-disparate sex^a^ with HIV prevalence; and (C) intergenerational sex^b^ with HIV prevalence among 12,730 sexually active AGYW accessing combination HIV services in Tanzania through the sauti project, 2015–2019^c^^,^^d^.

		Unadjusted PR (95% CI)	Adjusted PR (95% CI)	Weighted PR (95% CI)
A.	No transactional sex	1 (REF.)	1 (REF.)	1 (REF.)
	Transactional sex	1.41 (1.11, 1.80)	1.34 (1.04, 1.74)	1.28 (1.00, 1.63)
B.	No age-disparate sex	1 (REF.)	1 (REF.)	1 (REF.)
	Age-disparate sex	1.20 (0.94, 1.55)	1.13 (0.88, 1.46)	1.26 (0.99, 1.60)
C.	No intergenerational sex	1 (REF.)	1 (REF.)	1 (REF.)
	Intergenerational sex	1.09 (0.71, 1.67)	0.97 (0.64, 1.47)	1.11 (0.77, 1.60)

^a^ Defined as a sexual relationship with a male partner ≥5 years older than the participating AGYW.

^b^ Defined as a sexual relationship with a male partner ≥10 years older than the participating AGYW.

^c^ Among 12,730 AGYW who reported having ever engaged in sex, of whom 309 (2.4%) were missing measures of transactional sex and/or partner age and were excluded from analysis.

^d^ A directed acyclic graph (DAG) was used to identify a minimally sufficient adjustment set of covariates, and potential confounders were included based on prior literature. Confounders included in the adjustment set were age, adult support, marital status, prior pregnancy, food insecurity, early sexual debut, and survey version. For model A, intergenerational sex was also included as a potential confounder.

PR, prevalence ratio; CI, confidence interval.

### Older partners and HIV

There was a moderate association between age-disparate sex and HIV in unadjusted analysis (PR: 1.20; 95% CI 0.94, 1.55) that was attenuated when adjusted for potential confounders (PR: 1.15; 95% CI 0.89, 1.50). In weighted analysis, age-disparate sex was associated with a 26% increase in HIV prevalence (PR: 1.26; 95% CI 0.99, 1.60). Intergenerational sex was not strongly associated with HIV prevalence in unadjusted (PR: 1.09; 95% CI 0.71, 1.67), adjusted (PR: 1.00; 95% CI 0.65, 1.53), or weighted models (PR: 1.11, 95% CI 0.77, 1.60).

### Combined association of transactional sex and sex with older partners

In common referent analysis, AGYW who engaged in transactional sex in the absence of age-disparate partners had 1.41 (95% CI 1.02, 1.94) times the prevalence of HIV compared to AGYW who reported neither behavior (Table 3). Conversely, age-disparate sex was not associated with HIV prevalence in the absence of transactional sex (PR: 1.19, 95% CI 0.81, 1.67). The prevalence ratio for those who reported both transactional sex and age-disparate sex was 1.45 (95% CI 1.01, 2.08), which was less than the expected prevalence ratio using both additive and multiplicative criteria.

**Table 3:** Interaction between (a) transactional sex and age-disparate sex^a^ and (b) transactional sex and intergenerational sex^b^ on HIV prevalence among 12,730 sexually active AGYW in Tanzania, 2015–2019^c^.

	No transactional sex	Transactional sex
A. Age-disparate sex strata	Adjusted PR (95% CI)	Adjusted PR (95% CI)
No age-disparate sex	1.00 (REF)	1.41 (1.02, 1.94)
Age-disparate sex	1.16 (0.81, 1.67)	1.45 (1.01, 2.08)
B. Intergenerational sex strata	Adjusted PR (95% CI)	Adjusted PR (95% CI)
No intergenerational sex	1.00 (REF)	1.30 (1.00, 1.70)
Intergenerational sex	0.78 (0.40, 1.55)	1.55 (0.90, 2.65)

^a^ Defined as a sexual relationship with a male partner ≥5 years older than the participating AGYW.

^b^ Defined as a sexual relationship with a male partner ≥10 years older than the participating AGYW.

^c^ Confounders included in each adjustment set were age, adult support, marital status, prior pregnancy, food insecurity, early sexual debut, and survey version.

PR, prevalence ratio; CI, confidence interval.

### Subgroup analysis among AGYW age 15–19

Transactional sex was also common among AGYW age ≤19 (Table 4). Transactional sex, age-disparate sex, and intergenerational sex were not significantly associated with HIV prevalence among younger AGYW (Supplementary Table S2).

**Table 4:** Characteristics of 5,816 adolescent girls ages 15–19 accessing combination HIV prevention services in Tanzania through the Sauti project, 2015–2019^a^.

	Overall *N* = 5,816		No transactional sex *N* = 3,879 (68.2%)		Transactional sex *N* = 1,813 (31.9%)		
	Median	IQR	Median	IQR	Median	IQR	*p*-value
Age, years	18.0	17.0–19.0	18.0	17.0–19.0	18.0	17.0–19.0	<.001
** **	*n*	%	*n*	%	*n*	%	*p*-value
Married	553	9.7	320	8.4	219	12.3	<.001
Adult support^b^	3,015	53.5	1,758	46.5	1,230	68.9	<.001
Food insecurity^c^	1,735	30.9	899	23.8	820	46.2	<.001
Ever pregnant	1,321	22.7	679	17.5	627	34.6	<.001
Sexual debut <15 years	1,310	23.2	594	15.6	702	39.5	<.001
Sexual partner >5 years older	1,282	22.3	648	16.8	617	34.4	<.001
Sexually active, ever	4,272	74.3	2,399	61.9	1,800	100.0	<.001
Sexually active, last 12 months	3,576	63.7	1,913	50.3	1,618	92.1	<.001
Using modern contraception^d^	1,363	26.8	706	20.6	657	39.6	<.001
Condomless sex, last 12 months^e^	222	70.3	136	63.3	86	85.1	<.001
>1 partner, last 12 months	1,657	29.4	577	14.1	1,074	60.9	<.001
>2 partners, last 12 months	526	9.3	162	4.2	363	20.6	<.001
Sexual violence, ever	673	11.9	307	8.1	355	19.9	<.001
Sex with HIV + partner, last 12 months	163	2.9	76	2.0	8	4.6	<.001
Prevalent HIV	65	1.2	34	0.9	29	1.7	.002

^a^ Missing. Married: 103 (1.8%); adult support: 185 (3.2%); food insecurity: 202 (3.5%); pregnancy: 5 (0.1%); sexual debut: 180 (3.1%); partner age: 58 (1.0%); ever sexually active: 68 (1.2%); sexually active in the last year 204 (3.5%); number of sex partners: 174 (3.0%); sexual violence: 176 (3.0%); sex with HIV + partner: 165 (2.8%); HIV: 191 (3.3%).

^b^ Has an adult in the household or community who provides unconditional emotional and/ or financial support.

^c^ Could not afford to buy food or there was not enough food to eat at home.

^d^ Use of modern contraception asked on versions 1 and 2 of the index.

^e^ Used a condom never, almost never, or sometimes when having vaginal sex; asked on version 3 of the index.

## Discussion

In this study, we examined the relationship between HIV burden and transactional sex among over 14,000 AGYW accessing real-world HIV prevention services through the PEPFAR/USAID-funded Sauti Project in Tanzania. Transactional exchanges of money, services, or gifts for sex were common among AGYW enrolled in Sauti programing, including among girls as young as age 15. These exchanges occurred among AGYW who were mostly unmarried, had previously been pregnant, and were receiving emotional and financial support from older adults, including family and other community members. AGYW who reported transactional sex had an elevated burden of HIV, and transactional sex was strongly associated with HIV prevalence even in the absence of older partners. Given the multiple social and structural vulnerabilities that can lead to transactional sexual relationships, including those with older partners, there is need to identify HIV prevention approaches that are more responsive to AGYW who may necessitate tailored and targeted programmatic support early in adolescence.

The burden of HIV was not evenly distributed among AGYW in this analysis, with estimates of HIV prevalence the highest among young women who reported both transactional sex and sexual relationships with older partners. While intergenerational and transactional sex are often conceptualized in the literature as compatible risk factors for HIV acquisition (23, 24, 29, 48), few studies have explored the extent to which asymmetry in partner age and transactional sex interact to increase HIV-associated vulnerability (28). Consistent with our findings, one case-control study in South Africa found women who reported both transactional sex and intergenerational partners had nearly twice the odds of HIV compared to women who reported either experience alone (22). These findings contribute to a growing body of literature that aim to further distinguish heterogeneities of risk among AGYW and more effectively guide HIV prevention programming. Importantly, early findings from the DREAMS initiative have demonstrated little to no improvement in reducing HIV incidence across multiple countries in southern and eastern Africa (49). Our results suggest that aligning HIV prevention services with the needs of AGYW who necessitate more targeted and intense approaches- such as young women who engage in resource-driven transactional relationships with older partners- are needed to deliver meaningful reductions in incidence for AGYW moving forward. Approaches may include economic empowerment for broader social asset building (50), integrated family planning and maternal health services to support the needs of adolescent mothers (51), as well as vocational training to facilitate employment and improve income (52), among others.

Experiences of transactional sex are often predicated on structural vulnerabilities, gender norms, and power differentials that motivate AGYW to engage in resource-driven sexual relationships. Through the lens of social science and health disparities research, structural vulnerability generally refers to the intersection of local hierarchies and power dynamics that exacerbate individual health risks (53). The high burden of food insecurity, prior pregnancy, and sexual violence among AGYW in this study, particularly among those reporting transactional sex, reinforce the larger social and structural determinants of HIV that underly sexual exchanges and increase risk of infection. For AGYW living in poverty, transactional sex offers a means to provide food, clothing, shelter and other resources, and can establish pathways to support and security. These more explicit transactional relationships may be even riskier for younger AGYW, who may be further marginalized or restricted from accessing sexual and reproductive health services due to their age (54). While transactional sex and sex with older partners were not strongly associated with HIV prevalence among adolescents in subgroup analyses, an alarmingly high prevalence of condomless sex among girls as young as 15 engaged in transactional sex indicates a potential high incidence of infection in the context of these larger social and structural factors (7, 48, 55).

AGYW are broadly considered *a priori*ty population for HIV epidemic control, but not all AGYW have a high risk of acquiring HIV. While young women >18 years who identified as female sex workers were explicitly excluded from analyses, a high prevalence of condomless sex, violence, and food insecurity among adolescent girls accessing Sauti services suggests some overlap with sex work. Notably, few programs in southern and eastern Africa have evaluated the impact of tailored HIV programming for young women who sell sex (56–58). In Zimbabwe, DREAMS programming that implemented community mobilization and social protection interventions alongside dedicated HIV testing and PrEP for young women who sell sex found increased uptake of clinical services; however, no effect on HIV incidence was observed (59). Additional efforts in Zimbabwe, Mozambique, and Kenya have worked to engage young women who sell sex, but more broadly this population remains under-represented in both research and programs (58, 60). This gap may be further perpetuated by issues of identity, and young women who identify as sex workers face myriad stigmas that compromise their safety and wellbeing (61, 62). Moreover, young women who sell sex may not identify as sex workers until they are older (63, 64), but may be marginalized from existing youth-focused AGYW HIV prevention and treatment programs (16, 65, 66). They may also feel ostracized by older sex workers, thus limiting the extent to which they engage in FSW HIV prevention and treatment efforts such as drop-in centers which are not typically designed to serve youth. Thus, there is an urgent need to engage this population in both research and program design efforts, and to grow the evidence base of effective interventions for young women who participate in sexual-economic exchanges (67).

A strength of this study was our ability to leverage a large database of observational data collected during the delivery of routine services through the Sauti Project in Tanzania. In general, population-level inferences made using programmatic data tend to be more representative than research data, which generally are collected using narrow eligibility criteria that may threaten external validity (68). Well-controlled research studies may also exclude adolescents who are most at risk for poor sexual and reproductive health outcomes such as those who live and work on the street, potentially limiting generalizability of findings (69, 70). Yet programmatic data remain a relatively under-utilized resource to examine the effectiveness of HIV-related interventions and implementation strategies for key populations (71–74). As programs continue to roll out biomedical prevention interventions for AGYW such as long-acting PrEP, program data offer an opportunity to keep pace and quickly tailor and adapt implementation strategies, thus providing for a more nuanced and more efficient HIV response.

This study also had several limitations. Our use of program data necessitated the merging of multiple databases. Rigorous quality assurance procedures were implemented throughout the duration of the program; however, challenges with unique identifiers limited the number of AGYW who could be linked across program records. We used non-response weights to try and improve internal validity under the assumption these unlinked records were MAR given the observed patterns in which the “missingess” of records occurred. Additionally, changes to the vulnerability index and question format during program implementation precluded us from fully assessing key measures including frequency of condom use, which may have provided additional insight into behavioral risks within transactional sexual partnerships. Further, we were also limited by temporality and were unable to determine if transactional or intergenerational sexual partnerships were more recent, and whether HIV was sexually or pediatrically acquired. Nevertheless, these findings provide critical insights into multiple relationship dynamics among AGYW in Tanzania. Given relatively few research data available for AGYW in the context of HIV prevention and treatment uptake in Tanzania (75), these estimates fill an epidemiologic gap and can inform targets for intervention moving forward.

## Conclusions

Declines in HIV incidence among AGYW over the last several years have been smaller than expected given substantial investments in HIV programming for AGYW in eastern and southern Africa through large combination HIV prevention programs such as DREAMS. This study contributes to a growing body of evidence that HIV risks among AGYW are not homogenous, and that additional implementation strategies are needed to link AGYW at high-risk of HIV to behavioral, structural and biomedical prevention interventions such as PrEP. These efforts likely include increased specificity among AGYW programs to better meet the sexual and reproductive health needs of AGYW at high risk of HIV, but also investment in tailored youth-friendly strategies for AGYW who have been marginalized from the current HIV response, including young women who engage in sexual relationships with older partners and those who sell sex.

## Data Availability

The data analyzed in this study is subject to the following licenses/restrictions: Data may be made available upon reasonable written request to both the corresponding and senior authors. Requests to access these datasets should be directed to Katherine Rucinski, rucinski@jhu.edu and Kelly Curran, Kelly.Curran@jhpiego.org.

## References

[1] Joint United Nations Programme on HIV/AIDS. UNAIDS Data 2021. Geneva, Switzerland: UNAIDS (2021). Available online at: https://www.unaids.org/en/resources/documents/2021/2021_unaids_data (cited January 4, 2022)

[2] Saul J Bachman G Allen S Toiv NF Cooney C Beamon T. The DREAMS core package of interventions: a comprehensive approach to preventing HIV among adolescent girls and young women. PLOS One. (2018) 13(12):e0208167. 10.1371/journal.pone.0208167 PMC6285267 30532210

[3] Adolescents-HIV-Eastern-Southern-Africa-2021.pdf. Available online at: https://www.unicef.org/esa/media/8791/file/Adolescents-HIV-Eastern-Southern-Africa-2021.pdf (cited June 16, 2022)

[4] Tanzania Commission for AIDS (TACAIDS), Zanzibar AIDS Commission (ZAC). Tanzania HIV Impact Survey (THIS) 2016-2017: Final Report. Dar es Salaam, Tanzania. (2018). Available online at: https://phia.icap.columbia.edu/countries/tanzania/ (cited January 5, 2022)

[5] Brown K. Status of HIV epidemic control among adolescent girls and young women aged 15–24 years — seven African countries, 2015–2017. MMWR Morb Mortal Wkly Rep. (2018) 67(1):29–32. 10.15585/mmwr.mm6701a6 PMC5769792 29329280

[6] Harrison A Colvin CJ Kuo C Swartz A Lurie M. Sustained high HIV incidence in young women in Southern Africa: social, behavioral, and structural factors and emerging intervention approaches. Curr HIV/AIDS Rep. (2015) 12(2):207–15. 10.1007/s11904-015-0261-0 25855338 PMC4430426

[7] Comins CA Rucinski KB Baral S Abebe SA Mulu A Schwartz SR. Vulnerability profiles and prevalence of HIV and other sexually transmitted infections among adolescent girls and young women in Ethiopia: a latent class analysis. PLoS One. (2020) 15(5):e0232598. 10.1371/journal.pone.0232598 32407394 PMC7224533

[8] Delany-Moretlwe S Cowan FM Busza J Bolton-Moore C Kelley K Fairlie L. Providing comprehensive health services for young key populations: needs, barriers and gaps. J Int AIDS Soc. (2015) 18(2 Suppl 1):19833. 10.7448/IAS.18.2.19833 25724511 PMC4344539

[9] Pettifor AE Measham DM Rees HV Padian NS. Sexual power and HIV risk, South Africa. Emerg Infect Dis. (2004) 10(11):1996. 10.3201/eid1011.040252 15550214 PMC3328992

[10] Brook DW Morojele NK Zhang C Brook JS. South African adolescents: pathways to risky sexual behavior. AIDS Educ Prev. (2006) 18(3):259–72. 10.1521/aeap.2006.18.3.259 16774467

[11] Sanders RA. Adolescent psychosocial, social, and cognitive development. Pediatr Rev. (2013) 34(8):354–8. quiz 358–9. 10.1542/pir.34.8.354 23908362

[12] Jewkes RK Dunkle K Nduna M Shai N. Intimate partner violence, relationship power inequity, and incidence of HIV infection in young women in South Africa: a cohort study. Lancet. (2010) 376(9734):41–8. 10.1016/S0140-6736(10)60548-X 20557928

[13] Harrison A O’Sullivan LF Hoffman S Dolezal C Morrell R. Gender role and relationship norms among young adults in South Africa: measuring the context of masculinity and HIV risk. J Urban Health Bull N Y Acad Med. (2006) 83(4):709–22. 10.1007/s11524-006-9077-y PMC2430491 16758334

[14] Gottert A Barrington C McNaughton-Reyes HL Maman S MacPhail C Lippman SA Gender norms, gender role conflict/stress and HIV risk behaviors among men in Mpumalanga, South Africa. AIDS Behav. (2018) 22(6):1858–69. 10.1007/s10461-017-1706-9 28161801 PMC6440537

[15] Palermo T Chzhen Y Balvin N Kajula L Palermo T Groppo V Examining determinants of gender attitudes: evidence among Tanzanian adolescents. BMC Womens Health. (2020) 20(1):195. 10.1186/s12905-020-01057-8 32912210 PMC7488302

[16] Rucinski KB Schwartz SR Mishra S Phaswana-Mafuya N Diouf D Mothopeng T High HIV prevalence and low HIV-service engagement among young women who sell sex: a pooled analysis across 9 sub-Saharan African countries. J Acquir Immune Defic Syndr 1999. (2020) 85(2):148–55. 10.1097/QAI.0000000000002432 32639275

[17] Hokororo A Kihunrwa A Hoekstra P Kalluvya SE Changalucha JM Fitzgerald DW High prevalence of sexually transmitted infections in pregnant adolescent girls in Tanzania: a multi-community cross-sectional study. Sex Transm Infect. (2015) 91(7):473–8. 10.1136/sextrans-2014-051952 25834122 PMC4591089

[18] Erulkar A Medhin G Weissman E. The impact and cost of child marriage prevention in rural Tanzania. Addis Ababa: Population Council (2017). Available online at: https://knowledgecommons.popcouncil.org/departments_sbsr-pgy/536

[19] Topazian HM Stoner MCD Edwards JK Kahn K Gómez-Olivé FX Twine R Variations in HIV risk by young women’s age and partner age disparity in rural South Africa (HPTN 068). J Acquir Immune Defic Syndr 1999. (2020) 83(4):350–6. 10.1097/QAI.0000000000002270 PMC7722780 31904708

[20] de Oliveira T Kharsany ABM Gräf T Cawood C Khanyile D Grobler A Transmission networks and risk of HIV infection in KwaZulu-Natal, South Africa: a community-wide phylogenetic study. Lancet HIV. (2017) 4(1):e41–50. 10.1016/S2352-3018(16)30186-2 27914874 PMC5479933

[21] Okiring J Getahun M Gutin SA Lebu S Lee J Maeri I Sexual partnership concurrency and age disparities associated with sexually transmitted infection and risk behavior in rural communities in Kenya and Uganda. Int J Infect Dis. (2022) 120:158–67. 10.1016/j.ijid.2022.04.038 35472527 PMC9984205

[22] McCloskey LA Eloff I Doran K. Determinants of intergenerational sexual relationships and HIV risk among South African women outpatients in Gauteng. AIDS Care. (2021) 33(5):654–62. 10.1080/09540121.2020.1823311 32964726

[23] Kilburn K Ranganathan M Stoner MCD Hughes JP MacPhail C Agyei Y Transactional sex and incident HIV infection in a cohort of young women from rural South Africa. AIDS Lond Engl. (2018) 32(12):1669–77. 10.1097/QAD.0000000000001866 PMC6082595 29762176

[24] Jewkes R Dunkle K Nduna M Shai NJ. Transactional sex and HIV incidence in a cohort of young women in the stepping stones trial. J AIDS Clin Res. (2012) 3(5):1000158. 10.4172/2155-6113.1000158

[25] Wamoyi J Stobeanau K Bobrova N Abramsky T Watts C. Transactional sex and risk for HIV infection in sub-Saharan Africa: a systematic review and meta-analysis. J Int AIDS Soc. (2016) 19(1):20992. 10.7448/IAS.19.1.20992 27809960 PMC5095351

[26] Stoebenau K Heise L Wamoyi J Bobrova N. Revisiting the understanding of “transactional sex” in sub-Saharan Africa: a review and synthesis of the literature. Soc Sci Med. (2016) 168:186–97. 10.1016/j.socscimed.2016.09.023 27665064

[27] Wamoyi J Heise L Meiksin R Kyegombe N Nyato D Buller AM. Is transactional sex exploitative? A social norms perspective, with implications for interventions with adolescent girls and young women in Tanzania. PLoS One. (2019) 14(4):e0214366. 10.1371/journal.pone.0214366 30939145 PMC6445416

[28] Ranganathan M Kilburn K Stoner MCD Hughes JP MacPhail C Gomez-Olive FX The mediating role of partner selection in the association between transactional sex and HIV incidence among young women. J Acquir Immune Defic Syndr 1999. (2020) 83(2):103–10. 10.1097/QAI.0000000000002225 PMC6970545 31714368

[29] Stoner MCD Nguyen N Kilburn K Gómez-Olivé FX Edwards JK Selin A Age-disparate partnerships and incident HIV infection in adolescent girls and young women in rural South Africa. AIDS Lond Engl. (2019) 33(1):83–91. 10.1097/QAD.0000000000002037 PMC6279556 30289813

[30] Rucinski KB Kilburn K delong SM Gómez-Olivé FX Kahn K Twine R Developmental Trajectories of Transactional Sex and Age-Disparate Relationships During Adolescence: An HPTN 068 Analysis. Mexico City: Mexo (2019). 10.1007/s10461-024-04470-4 PMC12208780 39225891

[31] Kyegombe N Meiksin R Wamoyi J Heise L Stoebenau K Bulle AM. Sexual health of adolescent girls and young women in central Uganda: exploring perceived coercive aspects of transactional sex. Sex Reprod Health Matters. (2020) 28(1):1700770. 10.1080/26410397.2019.1700770 31934824 PMC7888006

[32] Becker ML Bhattacharjee P Blanchard JF Cheuk E Isac S Musyoki HK Vulnerabilities at first sex and their association with lifetime gender-based violence and HIV prevalence among adolescent girls and young women engaged in sex work, transactional sex, and casual sex in Kenya. J Acquir Immune Defic Syndr 1999. (2018) 79(3):296–304. 10.1097/QAI.0000000000001826 PMC6203425 30113403

[33] Wamoyi J Wight D Plummer M Mshana GH Ross D. Transactional sex amongst young people in rural Northern Tanzania: an ethnography of young women’s motivations and negotiation. Reprod Health. (2010) 7(1):2. 10.1186/1742-4755-7-2 20429913 PMC2867784

[34] Gichane MW Wamoyi J Atkins K Balvanz P Maman S Majani E The influence of cash transfers on engagement in transactional sex and partner choice among adolescent girls and young women in northwest Tanzania. Cult Health Sex. (2022) 24(1):1–15. 10.1080/13691058.2020.1811890 32935625

[35] Mbita G Mwanamsangu A Plotkin M Casalini C Shao A Lija G Consistent condom use and dual protection among female sex workers: surveillance findings from a large-scale, community-based combination HIV prevention program in Tanzania. AIDS Behav. (2020) 24(3):802–11. 10.1007/s10461-019-02642-1 31444713 PMC7018834

[36] Mbita G Komba AN Casalini C Bazant E Curran K Christensen A Predictors of HIV among 1 million clients in high-risk male populations in Tanzania. AIDS Behav. (2022) 26(10):1–14. 10.1007/s10461-022-03667-9 PMC9474353 35362905

[37] Nyato D Nnko S Komba A Kuringe E Plotkin M Mbita G Facilitators and barriers to linkage to HIV care and treatment among female sex workers in a community-based HIV prevention intervention in Tanzania: a qualitative study. PLoS One. (2019) 14(11):e0219032. 10.1371/journal.pone.0219032 31743336 PMC6863533

[38] Kuringe E Materu J Nyato D Majani E Ngeni F Shao A Prevalence and correlates of depression and anxiety symptoms among out-of-school adolescent girls and young women in Tanzania: a cross-sectional study. PLoS One. (2019) 14(8):e0221053. 10.1371/journal.pone.0221053 31419238 PMC6697336

[39] Wambura M Drake M Kuringe E Majani E Nyato D Casalini C Cash transfer to adolescent girls and young women to reduce sexual risk behavior (CARE): protocol for a cluster randomized controlled trial. JMIR Res Protoc. (2019) 8(12):e14696. 10.2196/14696 31859686 PMC6942193

[40] Han H Yang F Murray S Mbita G Bangser M Rucinski K Characterizing a sexual health and HIV risk stratification scale for sexually active adolescent girls and young women (AGYW) in Tanzania. PloS One. (2021) 16(3):e0248153. 10.1371/journal.pone.0248153 33735253 PMC7971553

[41] Leclerc-Madlala S. Age-disparate and intergenerational sex in Southern Africa: the dynamics of hypervulnerability. AIDS Lond Engl. (2008) 22(Suppl 4):S17–25. 10.1097/01.aids.0000341774.86500.53 19033752

[42] UNAIDS Terminology Guidelines—2015. Geneva: UNAIDS (2015). Available online at: https://www.unaids.org/en/resources/documents/2015/2015_terminology_guidelines (cited January 9, 2023)

[43] Knol MJ VanderWeele TJ. Recommendations for presenting analyses of effect modification and interaction. Int J Epidemiol. (2012) 41(2):514–20. 10.1093/ije/dyr218 22253321 PMC3324457

[44] Howe CJ Cole SR Lau B Napravnik S Eron JJ. Selection bias due to loss to follow up in cohort studies. Epidemiol Camb Mass. (2016) 27(1):91–7. 10.1097/EDE.0000000000000409 PMC5008911 26484424

[45] Cole SR Hernán MA. Constructing inverse probability weights for marginal structural models. Am J Epidemiol. (2008) 168(6):656–64. 10.1093/aje/kwn164 18682488 PMC2732954

[46] Stuart EA Azur M Frangakis C Leaf P. Multiple imputation with large data sets: a case study of the children’s mental health initiative. Am J Epidemiol. (2009) 169(9):1133–9. 10.1093/aje/kwp026 19318618 PMC2727238

[47] Azur MJ Stuart EA Frangakis C Leaf PJ. Multiple imputation by chained equations: what is it and how does it work? Int J Methods Psychiatr Res. (2011) 20(1):40–9. 10.1002/mpr.329 21499542 PMC3074241

[48] Gichane MW Rosenberg NE Zimmer C Pettifor AE Maman S Maseko B Individual and relationship-level correlates of transactional sex among adolescent girls and young women in Malawi: a multilevel analysis. AIDS Behav. (2022) 26(3):822–32. 10.1007/s10461-021-03442-2 34426863 PMC8840914

[49] Birdthistle I Kwaro D Shahmanesh M Baisley K Khagayi S Chimbindi N Evaluating the impact of DREAMS on HIV incidence among adolescent girls and young women: a population-based cohort study in Kenya and South Africa. PLoS Med. (2021) 18(10):e1003837. 10.1371/journal.pmed.1003837 34695112 PMC8880902

[50] Iwelunmor J Nwaozuru U Obiezu-Umeh C Uzoaru F Ehiri J Curley J Is it time to RE-AIM? A systematic review of economic empowerment as HIV prevention intervention for adolescent girls and young women in sub-Saharan Africa using the RE-AIM framework. Implement Sci Commun. (2020) 1(1):53. 10.1186/s43058-020-00042-4 32885209 PMC7427963

[51] Groves AK Maman S Stankard PH Gebrekristos LT Amon JJ Moodley D. Addressing the unique needs of adolescent mothers in the fight against HIV. J Int AIDS Soc. (2018) 21(6):e25155. 10.1002/jia2.25155 29956491 PMC6024120

[52] Kasirye R Laurenzi C Nabulya A Nakijoba B. Safe spaces, vocational training, and prevention programs protect young Ugandan women: findings from Uganda youth development link’s DREAMS initiative for rural communities. Vulnerable Child Youth Stud. (2023) 18(2):195–206. 10.1080/17450128.2022.2123118

[53] Bourgois P Holmes SM Sue K Quesada J. Structural vulnerability: operationalizing the concept to address health disparities in clinical care. Acad Med J Assoc Am Med Coll. (2017) 92(3):299–307. 10.1097/ACM.0000000000001294 PMC5233668 27415443

[54] Kangaude G Coast E Fetters T. Adolescent sexual and reproductive health and universal health coverage: a comparative policy and legal analysis of Ethiopia, Malawi and Zambia. Sex Reprod Health Matters. (2020) 28(2):1832291. 10.1080/26410397.2020.1832291 33121392 PMC7887923

[55] Gichane MW Moracco KE Pettifor AE Zimmer C Maman S Phanga T Socioeconomic predictors of transactional sex in a cohort of adolescent girls and young women in Malawi: a longitudinal analysis. AIDS Behav. (2020) 24(12):3376–84. 10.1007/s10461-020-02910-5 32405725

[56] Busza J Mtetwa S Mapfumo R Hanisch D Wong-Gruenwald R Cowan F. Underage and underserved: reaching young women who sell sex in Zimbabwe. AIDS Care. (2016) 28(Suppl 2):14–20. 10.1080/09540121.2016.1176673 27391994 PMC4991229

[57] Napierala S Chabata ST Fearon E Davey C Hargreaves J Busza J Engagement in HIV care among young female sex workers in Zimbabwe. J Acquir Immune Defic Syndr 1999. (2018) 79(3):358–66. 10.1097/QAI.0000000000001815 30036276

[58] Chabata ST Makandwa R Hensen B Mushati P Chiyaka T Musemburi S Strategies to identify and reach young women who sell sex with HIV prevention and care services: lessons learnt from the implementation of DREAMS services in two cities in Zimbabwe. JMIR Public Health Surveill. (2022) 8(7):e32286. 10.2196/32286 35896024 PMC9377473

[59] Chabata ST Hensen B Chiyaka T Mushati P Musemburi S Dirawo J The impact of the DREAMS partnership on HIV incidence among young women who sell sex in two Zimbabwean cities: results of a non-randomised study. BMJ Glob Health. (2021) 6(4):e003892. 10.1136/bmjgh-2020-003892 33906844 PMC8088246

[60] Bhattacharjee P Musau A Manguro G Ongwen P Mutegi J Kioko J HIV Prevention programme with young women who sell sex in Mombasa, Kenya: learnings for scale-up. J Int AIDS Soc. (2022) 25(8):e25969. 10.1002/jia2.25969 36028893 PMC9418418

[61] Lyons CE Schwartz SR Murray SM Shannon K Diouf D Mothopeng T The role of sex work laws and stigmas in increasing HIV risks among sex workers. Nat Commun. (2020) 11:773. 10.1038/s41467-020-14593-6 32071298 PMC7028952

[62] Chen C Baral S Comins CA Mcingana M Wang L Phetlhu DR HIV- and sex work-related stigmas and quality of life of female sex workers living with HIV in South Africa: a cross-sectional study. BMC Infect Dis. (2022) 22:910. 10.1186/s12879-022-07892-4 36474210 PMC9724359

[63] Hensen B Chabata ST Floyd S Chiyaka T Mushati P Busza J HIV risk among young women who sell sex by whether they identify as sex workers: analysis of respondent-driven sampling surveys, Zimbabwe, 2017. J Int AIDS Soc. (2019) 22(12):e25410. 10.1002/jia2.25410 31793748 PMC6887898

[64] Ranganathan M MacPhail C Pettifor A Kahn K Khoza N Twine R Young women’s perceptions of transactional sex and sexual agency: a qualitative study in the context of rural South Africa. BMC Public Health. (2017) 17:666. 10.1186/s12889-017-4636-6 28830394 PMC5568133

[65] Milovanovic M Jewkes R Matuludi M Dunkle K Hlongwane K Vanleeuw L Sex work and young women: a cross sectional study to understand the overlap of age and sex work as a central tenet to epidemic control in South Africa. AIDS Care. (2023) 35(4):1–9. 10.1080/09540121.2022.2057908 35373670

[66] Bowring AL Ketende S Rao A Njindam IM Decker MR Lyons C Characterising unmet HIV prevention and treatment needs among young female sex workers and young men who have sex with men in Cameroon: a cross-sectional analysis. Lancet Child Adolesc Health. (2019) 3(7):482–91. 10.1016/S2352-4642(19)30123-3 31105052

[67] Crankshaw TL Freedman J. Sex work or transactional sex? Shifting the dialogue from risk to rights. Sex Reprod Health Matters. (2023) 31(1):2210859. 10.1080/26410397.2023.2210859 37351919 PMC10291900

[68] Degtiar I Rose S. A review of generalizability and transportability. Annu Rev Stat Its Appl. (2023) 10(1):501–24. 10.1146/annurev-statistics-042522-103837

[69] Bekker. HIV and adolescents: focus on young key populations. J Int AIDS Soc. (2015) 18(2Suppl 1):20076. 10.7448/IAS.18.2.20076

[70] Cheuk E Isac S Musyoki H Pickles M Bhattacharjee P Gichangi P Informing HIV prevention programs for adolescent girls and young women: a modified approach to programmatic mapping and key population size estimation. JMIR Public Health Surveill. (2019) 5(2):e11196. 10.2196/11196 30932868 PMC6462887

[71] Rucinski K Masankha Banda L Olawore O Akolo C Zakaliya A Chilongozi D HIV testing approaches to optimize prevention and treatment for key and priority populations in Malawi. Open Forum Infect Dis. (2022) 9(4):ofac038. 10.1093/ofid/ofac038 35265725 PMC8900928

[72] Olawore O Astatke H Lillie T Persaud N Lyons C Kamali D Peer recruitment strategies for female sex workers not engaged in HIV prevention and treatment services in Côte d’Ivoire: program data analysis. JMIR Public Health Surveill. (2020) 6(4):e18000. 10.2196/18000 33001039 PMC7563635

[73] Ortblad KF Mawandia S Bakae O Tau L Grande M Mogomotsi GP Using routine programmatic data to measure HIV incidence among pregnant women in Botswana. Popul Health Metr. (2022) 20:10. 10.1186/s12963-022-00287-2 35246143 PMC8896233

[74] Rao A Mhlophe H Comins C Young K Mcingana M Lesko C Persistence on oral pre-exposure prophylaxis (PrEP) among female sex workers in eThekwini, South Africa, 2016–2020. PLoS One. (2022) 17(3):e0265434. 10.1371/journal.pone.0265434 35290421 PMC8923438

[75] Low A Gummerson E Schwitters A Bonifacio R Teferi M Mutenda N Food insecurity and the risk of HIV acquisition: findings from population-based surveys in six sub-Saharan African countries (2016–2017). BMJ Open. (2022) 12(7):e058704. 10.1136/bmjopen-2021-058704 35820770 PMC9277378

