# Supplementary material for: Transactional sex and age-disparate sexual partnerships among adolescent girls and young women in Tanzania
Source: Front Reprod Health. 2024 Jul 11;6:1360339. doi: 10.3389/frph.2024.1360339 (PMC11269161; doi:10.3389/frph.2024.1360339)
Supplement: Supplementary file 1 [file Table1.docx]

**S1 Table.** Characteristics of 65,185 AGYW accessing combination HIV prevention services in Tanzania through the Sauti Project, 2015-2019^a^

|  |  | No HIV testing record  n=50,312 | |  | Linked HIV testing record  n=14,873 | |  |  |
| --- | --- | --- | --- | --- | --- | --- | --- | --- |
|  |  | **Median** | **IQR** |  | **Median** | **IQR** | **p** |  |
| Age, years |  | 20.0 | 18.0-22.0 |  | 20.0 | 18.0-22.0 | <.001 |  |
|  |  | **n** | **%** |  | **n** | **%** | **p** |  |
| Married |  | 11,553 | 23.6 |  | 3,063 | 20.9 | <.001 |  |
| Adult support^b^ |  | 33,830 | 68.4 |  | 7,949 | 55.0 | <.001 |  |
| Food insecurity^c^ |  | 20,050 | 40.8 |  | 4,302 | 29.9 | <.001 |  |
| Ever pregnant |  | 21,472 | 42.7 |  | 6,035 | 40.7 | <.001 |  |
| Sexual debut <15 years |  | 9,856 | 20.3 |  | 2,544 | 17.7 | <.001 |  |
| Sexual partner >5 years older, ever |  | 13,802 | 28.0 |  | 4,162 | 28.3 | 0.444 |  |
| Sexual partner >10 years older, ever |  | 3,906 | 7.9 |  | 1,074 | 7.3 | 0.015 |  |
| Sexually active, ever |  | 43,813 | 87.6 |  | 12,730 | 86.4 | <.001 |  |
| Experienced sexual violence, ever |  | 10,734 | 21.8 |  | 1,901 | 13.1 | <.001 |  |
| Sexually active, last 12 months |  | 48,615 | 73.4 |  | 10,895 | 76.1 | <.001 |  |
| Using modern contraception^d^ |  | 14,375 | 44.9 |  | 4,836 | 36.8 | <.001 |  |
| Condomless sex^e^ |  |  |  |  |  |  |  |  |
| >1 partner, last 12 months |  | 19,691 | 40.2 |  | 5,014 | 34.8 | <.001 |  |
| >2 partners, last 12 months |  | 5,758 | 11.8 |  | 1,564 | 10.9 | 0.001 |  |
| Sex with HIV+ partner, last 12 months |  | 2,094 | 4.3 |  | 411 | 2.9 | <.001 |  |
| Transactional sex, ever |  | 22,626 | 45.1 |  | 5,490 | 37.0 | <.001 |  |

^a^ Missing. married: 1669 (2.6%); adult support: 1226 (1.9%); food insecurity: 1589 (2.4%); pregnancy: 84 (0.1%); sexual debut: 2298 (3.5%); partner age: 1087 (1.7%); ever sexually active: 435 (0.7%); sexual violence: 1401 (2.1%); contraception: 2682 (5.6%); number of sex partners: 2057 (3.2%); sex with HIV+ partner: 1738 (2.7%); transactional sex: 1011 (1.6%)

^b^ Has an adult in the household or community who provides unconditional emotional and/ or financial support

^c^ Could not afford to buy food or there was not enough food to eat at home

^d^ Asked only of participants who completed versions 1 and 2 of the vulnerability index

^e^ Used a condom never or almost never when having vaginal sex; asked only of participants who completed version 3 of the index
