# Supplementary material for: Transactional sex and age-disparate sexual partnerships among adolescent girls and young women in Tanzania
Source: Front Reprod Health. 2024 Jul 11;6:1360339. doi: 10.3389/frph.2024.1360339 (PMC11269161; doi:10.3389/frph.2024.1360339)
Supplement: Supplementary file 2 [file Table2.docx]

**S2 Table.** Unadjusted and adjusted associations of A) transactional sex with HIV prevalence; B) age-disparate sex^a^ with HIV prevalence; and C) intergenerational sex^b^ with HIV prevalence among 4,272 sexually active adolescent girls ages 15-19 accessing combination HIV services in Tanzania through the Sauti Project, 2015-2019^c,d^

|  |  | **Unadjusted PR (95% CI)** | **Adjusted PR (95% CI)** |
| --- | --- | --- | --- |
| A. | No transactional sex | 1 (REF.) | 1 (REF.) |
|  | Transactional sex | 1.67 (0.97, 2.89) | 1.48 (0.81, 2.69) |
| B. | No age-disparate sex | 1 (REF.) | 1 (REF.) |
|  | Age-disparate sex | 1.15 (0.65, 2.06) | 1.00 (0.55, 1.86) |
| C. | No intergenerational sex | 1 (REF.) | 1 (REF.) |
|  | Intergenerational sex | 1.20 (0.48, 3.02) | 1.09 (0.43, 2.76) |

Abbreviations. PR: prevalence ratio, CI: confidence interval

^a^ Defined as a sexual relationship with a male partner ≥5 years older than the participating AGYW.

^b^ Defined as a sexual relationship with a male partner ≥10 years older than the participating AGYW.

^c^ Among 4,272 adolescent girls who reported having ever engaged in sex, of whom 43 (1.0%) were missing measures of transactional sex and/or partner age and were excluded from analysis.

^d^ A directed acyclic graph (DAG) was used to identify a minimally sufficient adjustment set of covariates, and potential confounders were included based on prior literature. Confounders included in the adjustment set were adult support, marital status, prior pregnancy, food insecurity, early sexual debut, and survey version. For model A, intergenerational sex was also included as a potential confounder.
