# Supplementary material for: Transactional sex and age-disparate sexual partnerships among adolescent girls and young women in Tanzania
Source: Front Reprod Health. 2024 Jul 11;6:1360339. doi: 10.3389/frph.2024.1360339 (PMC11269161; doi:10.3389/frph.2024.1360339)
Supplement: Supplementary file 3 [file Datasheet1.docx]

**S1 File. SAUTI Adolescent Girls and Young Women Vulnerability Index**

**SAUTI AGYW VULNERABILITY INDEX**

**Introductory script to be read to each client before the interview starts:**

Hi, my name is *(name)* and I am a *(title)* for the *(title)* program.

I would like to ask you a few questions to help me advise you about services available to adolescent girls and young women in this community. These questions will take approximately 15 minutes to answer. I am aware that some of these questions are personal, so please be assured that I will not judge you in any way based on the answers you give. I have been trained to make sure that anything you tell me will remain strictly confidential. Your name will not be recorded. Your participation in this interview is voluntary and you can stop the interview at any time. Do you have any questions? *(Answer questions)*

Are you willing to participate? *(If yes)* Are you ready to begin now?

| **Part 1. Context Information** | | | | | |
| --- | --- | --- | --- | --- | --- |
|  | **Question** | | | | **Response** |
| 1 | Date of administration *(DD/MMM/YY e.g. 01.Aug.2017)* | | | | **\|__\|__\|.\|___\|___\|\|___\|___\|___\|___\|** |
| 2 | First Name of who administers questionnaire | | | |  |
| 3 | Last Name of who administers questionnaire | | | |  |
| 4 | Cadre: O EW O PE O Clinical staff  O Researcher O other, specify: _________________ | | | | |
| 5 | Participant’s unique identification number; **use following instructions:**  12 characters (FFSSRRRXDDYY)   - FF - Client’s **first** **name** – Last 2 letters - SS - Client’s **surname**– Last 2 letters - RRR - Region of birth – First 3 letters - X - sex code (1 for male, 2 for female) - DD- Date of birth (2 digits for day of birth e.g. 01 for 1st August 1977) - YY - Year of birth – last 2 digits | | | |  |
| 6 | a. SCAN QR CODE | | | | 7. TAKE GIS DATA |
| 8 | Region | | | |  |
| 9 | District | | | |  |
| 10 | Ward | | | |  |
| 11 | Village | | | |  |
| 12 | Venue | | 1. Home 2. Tent 3. SBCC groups 4. WORTH+ groups 5. Brothel/ Guests houses 6. Gheto 7. Bodaboda parking.) 8. Mine sites 9. Truck drivers sites 10. Bar/ local brews/ Night clubs 11. Fishing sites 12. Plantations 13. Other, specify…………………………. | |  |
| **Part 2. Respondent age** | | | | | |
| 1 | | Client date of birth **(dd/mm/yy)** | | __/___/__________  This client is [____\|____] years old  **If the participants is under 15 or over 24 years of age, end interview and thank her for her time** | |
| **Part 3. Respondent eligibility criteria – School status** | | | | | |
| 1 | | Are you currently enrolled in a school? | | 1. Yes: move on to next question 2. No: **ELIGIBLE**, **continue to PART 4** | |
| 2 | | Have you attended classes more than 10 days in the last three months, when not on public holidays or school vacation? | | 1. Yes: end interview and thank her for her time 2. No: **ELIGIBLE**, **continue to PART 4** | |
| **Only continue** in girls **not in school aged 15-19** and **all girls aged 20-24**  Otherwise end the interview and thank her for her time and availability. | | | | | |

| **Part 4. Risk Questions**  *For each question, circle the response and the corresponding number of points in the point column. Calculate the total score by adding all points circled together after you have completed the questionnaire***.** | | | |
| --- | --- | --- | --- |
|  | **Question** | **Response Categories** | **Points** |
| 1 | Are you currently married or living together with a man as if married?  If yes, how old were you when you got married/began living with a man as if married? | Married/began living with a man as if married at [__\|__] years old.  *(enter 66 if not married)* |  |
|  |  | 1. No, not married or living together with a man as if married. | 0 |
|  |  | 1. Yes, under 18 years old. | 3 |
|  |  | 1. Yes, 18-20 years old. | 2 |
|  |  | 1. Yes, older than 20 years. | 1 |
| 2 | Is there an adult in your household or community to whom you can go for emotional and/ or financial support if you need it without having to give something in return?  *(this refers to persons only and excludes structural programs)* | 1. Yes, both emotional and financial support. | 0 |
|  |  | 2. Yes, but only emotional support. | 2 |
|  |  | 3. Yes, but only financial support. | 2 |
|  |  | 4. No adult who supports me. | 3 |
| 3 | In the past four weeks, did you go to sleep at night hungry because you could not afford to buy food or there was not enough food for you to eat at home? If yes, how often did this happen? | 1. Not gone to sleep at night hungry in the past four weeks | 0 |
|  |  | 2. Yes, rarely  (Once or twice in the past four weeks). | 1 |
|  |  | 3. Yes, sometimes  (Three to ten times in the past four weeks). | 2 |
|  |  | 4. Yes, often  (More than ten times in the past four weeks). | 3 |
| 4 | Have you ever been pregnant? If yes, what age were you when you were first pregnant? | First pregnant at [__\|__] years old  *(enter 66 if never been pregnant)* |  |
|  |  | 1. Never been pregnant | 0 |
|  |  | 1. Yes, younger than 15 years old | 3 |
|  |  | 1. Yes, between 15 and 17 years old | 2 |
|  |  | 1. Yes, between 18 and 20 years old | 1 |
|  |  | 1. Yes, older than 20 years old | 1 |
| 5 | How old were you when you first had sex?  *(For the purposes of this interview, ‘sex’ is penetrative sex: when a male puts his penis inside of a* ***female’s vagina or anus****, including non-consensual sex such as rape.)* | [__\|__] years old  *(enter 66 if never had sex)* |  |
|  |  | 1. Never had sex in my life | 0 |
|  |  | 1. Younger than 15 years old | 3 |
|  |  | 1. Between 15 and 17 years old | 2 |
|  |  | 1. Between 18 and 20 years old | 1 |
|  |  | 1. Older than 20 years | 1 |
| 6 | Thinking about your current and past sexual partners, estimate the **biggest** ever age difference between you and anyone of the sexual partner?  *(Estimate age difference if exact age is not known and consider how old the girl was at this time)* | [__\|__] years age difference  *(enter 66 if never had sex)* |  |
|  |  | 1. The biggest age difference was less than 3 years | 0 |
|  |  | 1. Between 3 and 5 years | 1 |
|  |  | 1. Between 6 and 10 years | 2 |
|  |  | 1. Over 10 years | 3 |
|  |  | 1. Never had sex in my life   *(Double check with previous answer choices. Has the participant really never had sex previously?)* | 0 |
| 7 | Have you ever had vaginal sex, anal sex, or both?  *(For the purposes of this interview, ‘sex’ is penetrative sex, including non-consensual sex such as rape.)* | 1. Yes, vaginal sex only | 2 |
|  |  | 1. Yes, anal sex only | 3 |
|  |  | 1. Yes, both vaginal and anal sex | 3 |
|  |  | 1. Never had sex in my life   *(Double check with previous answer choice. Has the participant really never had sex previously?)* | 0 |
| 8 | In the last 12 months, have you had vaginal sex?  *(refer to calendar month, “e.g. between today and last year this month”)* | 1. No, I did not have vaginal sex within the past 12 months, but before. | 1 |
|  |  | 1. Yes, had vaginal sex at least once in the last 12 months | 2 |
|  |  | 1. Never had sex in my life   *(Double check with previous answer choices. Has the participant really never had sex previously?)* | 0 |
| 9 | Of the times you had vaginal sex in the last 12 months, how often did you use a condom?    *(This includes any penetrative sex irrespective of partner or relationship type or consensus and independent of time)* | 1. Never used a condom when having vaginal sex | 3 |
|  |  | 1. Almost never used a condom when having vaginal sex | 3 |
|  |  | 1. Sometimes used a condom when having vaginal sex | 2 |
|  |  | 1. Almost always used a condom when having vaginal sex | 1 |
|  |  | 1. Always used a condom when having vaginal sex | 0 |
|  |  | 1. I don’t know | 3 |
|  |  | 1. Never had sex in my life   *(Double check with previous answer choices. Has the participant really never had sex previously?)* | 0 |
| 10 | In the last 12 months, have you had anal sex?  *(refer to calendar month, “e.g. between today and last year this month”)* | 1. No, I did not have anal sex within the past 12 months, but before. | 1 |
|  |  | 1. Yes, had anal sex at least once in the last 12 months | 2 |
|  |  | 1. Never had sex in my life   *(Double check with previous answer choices. Has the participant really never had sex previously?)* | 0 |
| 11 | Of the times you had anal sex in the last 12 months, how often did you use a condom?    *(This includes any penetrative sex irrespective of partner or relationship type or consensus and independent of time)* | 1. Never used a condom when having anal sex | 3 |
|  |  | 1. Almost never used a condom when having anal sex | 3 |
|  |  | 1. Sometimes used a condom when having anal sex | 2 |
|  |  | 1. Almost always used a condom when having anal sex | 1 |
|  |  | 1. Always used a condom when having anal sex | 0 |
|  |  | 1. I don’t know | 3 |
|  |  | 1. Never had sex in my life   *(Double check with previous answer choices. Has the participant really never had sex previously?)* | 0 |
| 12 | In the last 12 months, what was the most number of sexual partners you have had during the same month (30-day period)?  *(includes any penetrative sex irrespective of partner or relationship type or frequency of sex with this partner)* | 1. One sexual partner | 1 |
|  |  | 2. Two sexual partners | 2 |
|  |  | 3. Three or more sexual partners | 3 |
|  |  | 4. Never had sex in my life  *(Double check with previous answer choices. Has the participant really never had sex previously?)* | 0 |
| 13 | Considering all of your sexual partners in the past 12 months, including current partners, do you know of their HIV status? Were they HIV+ or HIV-? | 1. None of the partner(s) is HIV+ positive | 1 |
|  |  | 1. There is at least one HIV + partner | 2 |
|  |  | 1. Don’t know the HIV status of all my partners | 3 |
|  |  | 1. Never had sex in my life   *(Double check with previous answer choices. Has the participant really never had sex previously?)* | 0 |
| 14 | Have you ever have had sex with anyone because you expected that he would provide you with gifts, help you to pay for things, or help you in other ways? | 1. No, had sex but without such expectations | 1 |
|  |  | 1. Yes, only gifts | 3 |
|  |  | 1. Yes, only for other services | 3 |
|  |  | 1. Yes, only for money | 3 |
|  |  | 1. Yes, for money, gifts or other services | 3 |
|  |  | 1. Refuse to answer | 3 |
|  |  | 1. Never had sex in my life   *(Double check with previous answer choices. Has the participant really had never had sex previously?)* | 0 |
| 15 | At any time in your life, as a child or as an adult, have you ever experienced sexual violence? If yes, how often have you experienced this kind of sexual violence?  *(For the purposes of this survey, ‘sexual violence’ is any physical sexual act that is perpetrated against your will (this includes, for example vaginal or anal penetration).* | 1. Never experienced sexual violence | 0 |
|  |  | 1. Yes, once | 1 |
|  |  | 1. Yes, two times | 2 |
|  |  | 1. Yes, three times or more | 3 |
|  |  | **TOTAL SCORE** |  |
| **5. Total risk score category (circle only one that applies)**   1. Very high (32-43 or more than 3 concurrent partners OR sex for good OR sex for cash) 2. High (22-31) 3. Medium (11-21) 4. Low (0-10)   **If the client answered in any of the following ways, THEN SHE IS CATEGORIZED AS VERY HIGH VULNERABLE, REGARDLESS OF THE TOTAL SCORE**   - Question 12, Response 3 (3 or more sexual partners) - Question 14, Responses 2, 3 or 4 (Sex in exchange for gifts/ services, cash or both) | | | |
